# Endovascular occlusion of external carotid artery: internal jugular vein fistula via direct transcranial access to transverse-sigmoid sinus

**DOI:** 10.3171/2025.7.FOCVID2537

**Published:** 2025-10-01

**Authors:** Kautilya R. Patel, William E. Thorell, Nicholas Borg, Frank M. Mezzacappa, Carlos M. Alvarez, Daniel L. Surdell, Mithun G. Sattur

**Affiliations:** Department of Neurosurgery, University of Nebraska Medical Center, Omaha, Nebraska

**Keywords:** arteriovenous fistula, external carotid artery, internal jugular vein, transverse-sigmoid sinus, endovascular embolization, transcranial

## Abstract

Endovascular transarterial or retrograde transvenous occlusion is the preferred modality for treatment of dural arteriovenous fistulas at most intracranial locations. However, transvenous navigation to the segment of interest might not be possible due to occlusion or stenosis of an extracranial or intracranial major venous channel. Direct transcranial access to a major venous sinus proximal to the occlusion is a useful strategy in such situations. This video demonstrates successful endovascular coil embolization of an external carotid artery–internal jugular vein fistula below the jugular foramen through direct transcranial access to the transverse-sigmoid sinus in a patient with bilateral internal jugular vein occlusion.

The video can be found here: https://stream.cadmore.media/r10.3171/2025.7.FOCVID2537

## Transcript

In this video, we describe a hybrid transcranial endovascular transvenous occlusion of a high cervical internal jugular vein fistula with severe intracranial hypertension.

### 0:34 Patient Presentation.

Our patient was a gentleman in his 50s, who 8 months prior to current presentation developed eye redness and retro-orbital fullness. He was evaluated by ophthalmology with findings of papilledema and normal visual acuity. The imaging at that time was interpreted as normal. Opening pressure on lumbar puncture was 41 cm H_2_O, and he was treated with acetazolamide for 8 months, with symptom improvement and improvement in papilledema. His current outpatient exam showed marked worsening in papilledema, and he was referred for urgent management. Surprisingly, his visual symptoms remained minimal. Opening pressure on lumbar puncture on admission was 55 cm H_2_O.

### 1:17 Past History.

He had a significant past medical history of squamous cell carcinoma of the right tonsil that was treated 5 years ago with chemoradiation, as also squamous cell carcinoma of the left half of the tongue treated 2 years ago with surgery and limited neck dissection.

### 1:35 Imaging.

On the current admission, he was investigated with a series of imaging. The image on the top left shows the MRI brain with empty sella and tortuous optic nerves suggestive of intracranial hypertension. The lower left image is an axial time-of-flight MRA. At the skull base on the right side, several small vessels are apparent between the right internal carotid artery and the signal of the right internal jugular vein. The right transverse-sigmoid sinus system is opacifying and the left only minimally so. The lower right image is a coronal CT angiogram showing opacification of only the cranial portion of the right internal jugular vein and the lack of opacification of the left internal jugular vein.

### 2:20 Angiography.

Catheter angiography was performed via a right radial artery approach. Selective right external carotid artery angiogram showed a high-flow fistula between the right occipital artery and the cranial segment of the right internal jugular vein with retrograde reflux of blood into the cranium via the transverse-sigmoid system. The AP view of the right external carotid artery angiogram showed dramatic retrograde reflux intracranially of arterialized blood to the level of the superior sagittal sinus. The lateral view of the right internal carotid artery angiogram showed striking lack of utilization of antegrade sagittal sinus venous outflow. Instead, most of the venous outflow is occurring via prominent anterior skull base extra-jugular collaterals and some scalp vein collaterals. AP view of the right internal carotid artery angiogram and the left carotid injection showed similar findings. AP view of the vertebral artery angiogram correspondingly also shows venous congestion in the posterior fossa.

### 3:25 Pathophysiology.

Therefore, our hypothesis was that the patient suffered from radiation-induced high-flow fistula between the cranial segment of the internal jugular vein and external carotid artery, which, coupled with right internal jugular vein occlusion secondary to radiation, led to a high-flow reflux and retrograde flow of arterialized blood into the intracranial venous sinuses.^1^ Along with occlusion of the left internal jugular vein, possibly related to the prior neck dissection, these together led to severe intracranial congestion.

### 3:58 Treatment Options: Surgical Ligation.

We systematically considered several surgical and endovascular options for treatment of this fistula. Surgical neck dissection and fistula occlusion is a direct focal approach; however, it is fraught with difficulty due to postradiation surgical scarring and obscuration of surgical planes. The high cervical location might also necessitate mandible dislocation, and there is a high risk of cranial nerve palsy and neck hematoma.

### 4:25 Treatment Options: Endovascular.

Transarterial embolization either via the transradial or transfemoral route is a straightforward familiar approach to most endovascular practitioners. But due to the myriad of arterial feeders into the fistula, complete elimination was estimated to be difficult. A transvenous transfemoral endovascular route would be a potentially elegant solution to achieve fistula occlusion, but we were faced in this particular case with the issue of an occluded internal jugular vein toward the root of the neck, as also occlusion of the left internal jugular vein that precluded contralateral access. We also entertained a direct percutaneous transcervical access to the high cervical fistula segment; however, we were concerned with postradiation cutaneous changes that had rendered the skin hypertrophic and very thick, as also the risk of formation of a neck hematoma.

### 5:23 Treatment Options: Transcranial Transvenous Sinus Embolization.

Finally, we entertained the option of a transcranial direct venous sinus access to the transverse-sigmoid junction and thereby antegrade endovascular access through the shortest route to the site of the fistula.^2–6^ The disadvantages would be the requirement for a burr hole or a mini-craniotomy, with the higher risk of potential bleeding from arterialized flow in the sinus. We were also considering the risks of postoperative hematoma and, logistically, the need for either a hybrid operating room or the need to transport the patient between the operating room and the interventional angiography suite.

### 6:05 Initial Attempts and Final Plan.

Indeed, initial attempts including transfemoral transvenous, transradial transarterial, and percutaneous attempts proved unsuccessful. Following these initial attempts that were unsuccessful, we discussed with the patient and decided to proceed with transcranial exposure of the transverse-sigmoid junction, cannulation of the venous sinus, and direct endovascular access to the high cervical internal jugular vein, followed by fistula occlusion with coil embolization.

### 6:37 Surgery Setup.

The surgery was planned with general anesthesia and endotracheal intubation. The position was supine with the head turned to the left side with the head fixed in three-pin Mayfield head clamp fixation. Intraoperative adjuncts used were frameless stereotactic neuronavigation to localize the transverse-sigmoid junction, intraoperative Doppler to insonate the sinus after exposure, and fluoroscopy for catheterization of the sinus. We did not have access to a hybrid operating room, and therefore the initial portion of the procedure was performed in the operating room.

### 7:11 Procedure: Operating Room.

Intraoperative pictures show a curvilinear incision marked in the right retroauricular region spanning the transverse-sigmoid junction. Following incision of the skin and exposure of the underlying skull, the frameless stereotactic neuronavigation probe was used to confirm location of the transverse-sigmoid junction at the asterion. A single burr hole was placed and enlarged. We then used the intraoperative Doppler to confirm location of the sinus. The C-arm fluoroscopy unit was brought into the field.

We then performed micropuncture access of the venous sinus with return of arterialized blood. The guidewire was then passed into the transverse sinus, sigmoid sinus, jugular bulb, and the high cervical internal jugular vein. We then placed a 4-Fr micropuncture sheath into the venous sinus. The sheath was secured to the surrounding skin with a suture. We confirmed the free return of blood, and the sheath was further reinforced with adhesive dressing to prevent dislodgment during transfer to the angiography suite. Heparinized saline flush was hooked up to the sheath during transport from the operating room to the angiography suite.

### 8:20 Procedure: Angiography Suite.

In the angiography suite, we reconfirmed sheath placement using contrast injection. We then inserted a 0.017-inch microcatheter over a 0.014-inch microwire. We then progressively performed coil embolization of the fistulous segment. We also obtained right radial artery access and placement of a diagnostic catheter in the right common carotid artery to obtain control runs. Progressive coil embolization through the microcatheter was achieved with formation of a good coil mass in the fistulous segment and complete elimination of fistulous flow on control angiograms. The micropuncture sheath was withdrawn and hemostasis was achieved with Gelfoam. The incision was closed in standard fashion in the angiography suite.

### 9:03 Postoperative Course.

Postoperative course was uneventful, and he was discharged home on day 2. At follow-up at 3 months, he had significant improvement in papilledema and complete resolution at 6 months’ follow-up. He is completing a tapering regimen of acetazolamide.

### 9:17 Follow-Up Angiogram.

Three months’ follow-up angiogram was performed, and right external carotid injection showed complete fistula elimination. The AP view showed that there was no longer reflux of intracranial retrograde flow of arterialized blood. The lateral internal carotid artery angiogram showed that there was now antegrade venous sinus flow in the sagittal sinus, but because there was bilateral internal jugular vein occlusion, the ultimate cranial venous outflow was still occurring through the collateral venous channels.

A similar appearance was seen on left carotid artery angiogram where there was antegrade venous sinus flow. The vertebral artery angiogram also showed resolution of the venous hypertension and robust antegrade venous sinus drainage. Thus, the high-grade fistula was completely eliminated. While such a hybrid approach is rarely required, it is a strategic tool that can be successful in elimination of certain types of dural fistulas.

## Acknowledgments

We thank Mrs. Kimberly Peeples for creating the illustration (presented in the video) demonstrating the procedure.

## Disclosures

Dr. Thorell reported consulting fees from Progressive Neuro outside the submitted work.

## Author Contributions

Primary surgeon: Sattur. Assistant surgeon: Borg, Mezzacappa, Alvarez. Editing and drafting the video and abstract: Sattur, Patel. Critically revising the work: Sattur, Patel, Thorell, Borg, Mezzacappa, Alvarez. Reviewed submitted version of the work: all authors. Approved the final version of the work on behalf of all authors: Sattur. Supervision: Sattur. Case preparation with primary surgeon: Surdell.

## Supplemental Information

### Patient Informed Consent

The necessary patient informed consent was obtained in this study.
